# *MC03g0810*, an Important Candidate Gene Controlling Black Seed Coat Color in Bitter Gourd (*Momordica* spp.)

**DOI:** 10.3389/fpls.2022.875631

**Published:** 2022-04-27

**Authors:** Jian Zhong, Jiaowen Cheng, Junjie Cui, Fang Hu, Jichi Dong, Jia Liu, Yichao Zou, Kailin Hu

**Affiliations:** ^1^College of Horticulture, South China Agricultural University, Guangzhou, China; ^2^Key Laboratory of Biology and Genetic Improvement of Horticultural Crops (South China), Ministry of Agriculture and Rural Affairs, Guangzhou, China; ^3^Guangdong Vegetables Engineering Research Center, Guangzhou, China; ^4^Department of Horticulture, Foshan University, Foshan, China; ^5^Henry Fok School of Biology and Agricultural, Shaoguan University, Shaoguan, China

**Keywords:** bitter gourd, seed coat color, bulked segregant analysis, linkage analysis, fine-mapping

## Abstract

Seed coat color is one of the most intuitive phenotypes in bitter gourd (*Momordica* spp.). Although the inheritance of the seed coat color has been reported, the gene responsible for it is still unknown. This study used two sets of parents, representing, respectively, the intersubspecific and intraspecific materials of bitter gourd, and their respective F_1_ and F_2_ progenies for genetic analysis and primary mapping of the seed coat color. A large F_2:3_ population comprising 2,975 seedlings from intraspecific hybridization was used to fine-map the seed coat color gene. The results inferred that a single gene, named *McSC1*, controlled the seed coat color and that the black color was dominant over the yellow color. The *McSC1* locus was mapped to a region with a physical length of ∼7.8 Mb and 42.7 kb on pseudochromosome 3 *via* bulked segregant analysis with whole-genome resequencing (BSA-seq) and linkage analysis, respectively. Subsequently, the *McSC1* locus was further fine-mapped to a 13.2-kb region containing only one candidate gene, *MC03g0810*, encoding a polyphenol oxidase (PPO). Additionally, the variations of *MC03g0810* in the 89 bitter gourd germplasms showed a complete correlation with the seed coat color. Expression and PPO activity analyses showed a positive correlation between the expression level of *MC03g0810* and its product PPO and the seed coat color. Therefore, *MC03g0810* was proposed as the causal gene of *McSC1*. Our results provide an important reference for molecular marker-assisted breeding based on the seed coat color and uncover molecular mechanisms of the seed coat color formation in bitter gourd.

## Introduction

The seed coat color is one of the most important agronomic traits in many crops. Seeds with colored coats have higher nutritional and healthy values (Zhu, 2018), for instance, yellow coat seeds of Brassicaceae crops have a lower lignin concentration, thinner shells, less fiber, and higher amounts of oil and protein than the black ones (Marles and Gruber, 2004; Yu et al., 2007). The seed coat pigmentation of soybean (*Glycine max*) affects the levels of isoflavones and fatty acids, with the black varieties exhibiting the highest antioxidant activity compared with the other varieties (Cho et al., 2013; Lee et al., 2017). The seed coat color is also closely related to the seed viability, weight, and germination rate (Mavi, 2010). Additionally, dark-colored seeds have higher resistance to various pathogens and herbivores than those with light color, probably because higher polyphenol oxidase activity in the former enhances their stress tolerance (Marles et al., 2008; Fuerst et al., 2014) and the dark color is more likely to form a natural camouflage to prevent feeding by birds (Zhu et al., 2011).

In Cucurbitaceae crops, the seed coat displayed a wide range of colors. For example, *Luffa acutangula* and *Luffa cylindrica* have black seed coats, *Bryonia alba*, *Diplocyclos palmatus*, and *Nothoalsomitra suberosa* have brown, while *Acanthosicyos horridus* and *Lagenaria spaerica* have whitish yellow, and other cucurbits mostly have yellow to brown seed coats (Heneidak and Khalik, 2015). Each cucurbit has distinct seed features, for example, watermelon (*Citrullus lanatus*) seed coats are flat black, dotted black, green, tan, clump, red, white with tan tip, or white with pink tip (McKay, 1936; Poole et al., 1941). Therefore, Cucurbitaceae crops are ideal for studying the inheritance of the seed coat color.

The seed coat color is genetically controlled in Cucurbitaceae. In watermelon, a four-gene model, including *R*, *T*, *W*, and *D* genes, was proposed to elucidate the inheritance of the seed coat color, of which gene *D* determines the phenotype only when the other three genes are in the dominant state (Poole et al., 1941). In addition, *T^1^*, different from the *T* locus, was reported to produce dotted black (*RT*^1^*W*, *Rt*^1^*W*) and tan (*rT*^1^*W*) seed coats (Paudel et al., 2019). Recently, the *R*, *T^1^*, *W*, and *D* genes have been mapped on chromosomes 3, 5, 6, and 8, respectively, based on the QTL-seq analysis (Paudel et al., 2019). Furthermore, *Cla019481*, encoding a polyphenol oxidase (PPO) protein, was proposed as an important candidate for the *W* gene responsible for the black seed coat formation in watermelon (Li et al., 2018, 2020). The white seed coat color in pumpkin (*Cucurbita maxima*) is controlled by a single gene, *wsc*, which is recessive to the yellow seed coat color (Shi et al., 2021). A candidate gene (*CmaCh15G005270*) encoding an MYB transcription factor has been recently proposed for the *wsc* gene using the fine-mapping approach (Shi et al., 2021). In melon (*Cucumis melo*), a single dominant gene, *CmSC1*, controls the white seed coat color that is dominant over the yellow color (Ma et al., 2021). Conversely, the yellow seed coat color is controlled by a single dominant gene, *CmBS-1*, and is dominant over the brown color (Hu et al., 2021). Recently, *MELO3C014406*, a melon orthologous gene of the *Arabidopsis AtTT8* encoding a basic helix–loop–helix domain protein, has been identified as a candidate gene of the *CmSC1* (Ma et al., 2021). The *MELO3C019554*, encoding a homeobox protein, has been proposed as a candidate gene of *CmBS-1 via* the fine-mapping strategy (Hu et al., 2021).

Bitter gourd (*Momordica* spp.), an herbaceous climbing plant of Cucurbitaceae, originated from the tropical regions of Africa and is currently widely distributed in tropical and subtropical regions of Africa and Asia (Schaefer and Renner, 2010). Its seeds have various bioactive components such as ribosome-inactivating proteins (Puri et al., 2009), trypsin inhibitors (Lo et al., 2014), cucurbitane triterpenoids (Chen et al., 2005; Shah et al., 2014), and hypoglycemic peptide (Khanna et al., 1981; Wang et al., 2011). Most cultivated bitter gourds have yellow seeds; however, black, brown patched, brownish tan, and whitish brown-colored seeds have also been reported in the wild and semi-domesticated varieties (Kole et al., 2020). Genetic analysis indicated that the black seed coat color of the bitter gourd is dominant over the yellow one, thus conforming to the genetic model of a single dominant gene (Liu, 2011; Tan et al., 2013). In addition, the black coloration was dominant over the creamy seed coat color but exhibited a digenic inheritance mode. Furthermore, one of two loci was mapped on LG3 between the amplified fragment length polymorphism (AFLP) markers, E12M47a and E11M48a, which were 52.5 cM apart (Kole et al., 2012). A region spanning ∼300 kb on pseudochromosome 3 was reported to be related to the seed coat color through a genome-wide association study (Cui et al., 2022). To our knowledge, there is no further study on the mining of the seed coat color genes in bitter gourd until now.

Thus, this study aimed at identifying the gene regulating the black seed coat color in bitter gourds. We used intersubspecific and intraspecific F_2_ populations for the genetic analysis to ensure the inheritance reliability of the seed coat color in bitter gourd. Forward genetics strategies, including BSA-seq, linkage analysis, and fine-mapping, were used to identify genes regulating the seed coat color in the bitter gourd. Our study provides a basis for molecular marker-assisted breeding and understanding the molecular mechanisms of the seed coat color formation in bitter gourd.

## Materials and Methods

### Plant Materials

Four inbred lines of bitter gourd were used in this study. These included two lines with yellow seed coats, FOLI112 (*Momordica charantia* ssp. *charantia*) and S156 (*M. charantia* ssp. *charantia*), and another two lines with black seed coats, THMC170 (*M. charantia* ssp. *macroloba*) and 8–201 (*M. charantia* ssp. *charantia*). Among them, FOLI112 is a gynoecious line, which is a near-isogenic line of Dali-11, whose whole genome sequence is available (Cui et al., 2020), while THMC170 belongs to a variety with elongated seeds and small fruits. All the four inbred lines have completed resequencing. An intersubspecific F_1_ generation and F_2_ segregating population consisting of 120 individuals were constructed through artificial hybridization and self-pollination using FOLI112 and THMC170 as female and male parents, respectively. Similarly, an intraspecific F_1_ generation and F_2_ segregating population comprising 147 individuals were constructed using S156 and 8–201 as female and male parents, respectively. The two sets of F_1_ and F_2_ and their respective parental lines were then used for inheritance analysis of the seed coat color. Subsequently, the intersubspecific F_2_ population and its parental lines were used for BSA-seq analysis, and the intraspecific F_2_ population was used for linkage analysis. A large F_2:3_ population consisting of 2,975 seedlings self-crossed from several intraspecific F_2_ individuals was used for screening the recombinants *via* a fine-mapping strategy. Additionally, 89 bitter gourd materials, including 58 inbred lines and 31 F_1_ hybrids (Supplementary Table 1), were selected for cloning and comparing the candidate gene controlling the seed coat color. All plant materials, including the recombinants, were cultivated in an experimental field at the South China Agriculture University Teaching and Research Base in Zengcheng District, Guangzhou (23.24N, 113.64E).

### Bulked Segregant Analysis With Whole-Genome Resequencing

The DNA of all samples was extracted using the CTAB method (Porebski et al., 1997). For BSA-seq, yellow and black DNA pools were constructed by sampling equal amounts of young fresh leaf tissues from each of the 30 individuals with yellow and black seed coats from the intersubspecific F_2_ population.

Sequencing libraries of the yellow and black DNA pools and their parental DNA pools were prepared using the AxyPrep Mag PCR clean-up kit, according to the manufacturer’s instructions (Illumina). The generated sequences were further amplified using 150 bp paired-end sequencing on an Illumina X-ten system. Raw reads were filtered by removing low-quality reads containing adapters, unknown nucleotides (N) of more than 10%, and low-quality (*Q*-value ≤ 20) bases of more than 50%. Subsequently, clean reads were aligned to the Dali-11 reference genome (Cui et al., 2020) using Burrows–Wheeler Aligner (Li and Durbin, 2009), and the alignment files were converted to SAM/BAM files using SAMtools (Li H. et al., 2009). The Genome Analysis Toolkit was used for multi-sample variant calling, and its VariantFiltration tools with uniform standards (Windows 4, filter “QD < 4.0| | FS > 60.0 | | MQ < 40.0,” G_filter “GQ < 20”) were used to filter single nucleotide polymorphisms (SNPs) and insertions–deletions (InDels) (McKenna et al., 2010). We used ANNOVAR software (Wang et al., 2010) for aligning and annotating the SNPs and InDels to determine the physical positions of each variant.

Frequency distributions of each SNP (SNP-index) in the DNA pool were analyzed by the sliding window analysis using a window size of 1,000 kb and a step length of 100 kb. The SNPs with SNP-index values less than 0.3 or greater than 0.7 and a read depth of less than 7 were excluded. The ΔSNP-index was calculated by subtracting the SNP-indexes between the yellow and black DNA pools. Finally, the initial location of the genes regulating the seed coat color was identified using the positive or negative peak regions with a 95 and 99% confidence interval in 10,000 bootstrap replicates.

### Linkage Analysis

InDel primer pairs in the 99% confidence interval of BSA-seq based on the intersubspecific materials were designed by Primer 3 software, based on alignment results of S156 and 8–201 to the Dali-11 reference genome (Cui et al., 2020) aligned by SOAP2 (Li R. et al., 2009). Moreover, the polymerase chain reaction (PCR) was performed in a 10 μL reaction mixture containing 50–100 ng of template DNA, 0.2 μL of forward and reverse primers (10 μmol/L), and 5 μL of Green Taq Mix (Vazyme, Nanjing, China) for identifying the polymorphic InDel markers between the parental lines S156 and 8–201. The PCR conditions were as follows: an initial denaturation cycle of 3 min at 94°C; 34 cycles of denaturation at 94°C for 15 s, annealing at 55°C for 15 s, and extension at 72°C for 30 s; and a final extension cycle of 5 min at 72°C. The PCR products were then genotyped in 6% polyacrylamide gel electrophoresis (PAGE). Subsequently, linkage analysis was performed using the JoinMap 4.0 (Van Ooijen, 2006). Additionally, the seed coat color of each F_2_ plant was converted into genotypic codes (A/C), and the linkage groups were established using the independence logarithm of the odds (LOD). The LOD parameter used had a LOD-score threshold of 3.0 and a recombination frequency smaller than 0.4. A regression algorithm was used for mapping, and recombination values were converted to genetic distances using Haldane’s mapping function.

### Fine-Mapping

A large F_2:3_ population consisting of 2,975 seedlings self-crossed from several S156 × 8–201 F_2_ individuals was used to screen recombinants. These S156 × 8–201 F_2_ plants had sequential heterozygosity between SC_7 and SC_12, two flanking markers of co-segregation interval based on the linkage analysis. Thereafter, a precise interval was identified based on phenotypes and genotypes of recombinant plants.

### Gene Cloning and Sequence Analyses

The sequences of primer pairs used for cloning the gene encoding the seed coat color are listed in Supplementary Table 2. The PCR was performed in a 20 μL mixture using Phanta Max Super-Fidelity DNA Polymerase (Vazyme, Nanjing, China) according to the manufacturer’s protocol. Subsequently, all PCR products were purified and cloned into the pMD™19-T vector (Takara, Japan). Randomly selected positive colonies (at least three) for each amplicon were sequenced and assembled. Sequence alignment was performed using Clustal Omega,^1^ and the Conserved Domain tool from NCBI^2^ was used for the conserved domain analysis.

### Expression Analyses

Tissues from S156 and 8–201 lines were excised and frozen in liquid nitrogen for total RNA extraction. These tissues included root, stem, leaf, ovary, and sarcocarp at 18 days after flowering (DAF) and seed coat and embryo at three different developmental stages (12, 18, and 24 DAF). The Eastep Super Total RNA Extraction Kit (Promega, Shanghai, China) and the Eastep RT Master Mix Kit (Promega, Shanghai, China) were used for total RNA isolation and synthesis of the first cDNA strand, respectively, according to the manufacturer’s protocol. The real-time quantitative PCR (qRT-PCR) was carried out on a CFX384 Real-Time System (Bio-Rad, CA, United States) using TB Green^®^ Premix Ex Taq*™* II (Takara Japan). The relative expression levels with three biological and technical replicates for each sample were calculated using the delta–delta Ct method (2^–△△Ct^) (Livak and Schmittgen, 2001). The primer sequences of expression analysis are provided in Supplementary Table 2.

### Polyphenol Oxidase Activity Analyses

The seed coats were collected from the parental lines S156 and 8–201 at 18 and 24 DAF, respectively, and were immediately snap-frozen in liquid nitrogen. The bicinchoninic acid (BCA) kit (Elabscience, Wuhan, China) was used to extract crude enzymes and determine total protein concentration. Subsequently, PPO activity was evaluated using the PPO Colorimetric Assay Kit (Elabscience, Wuhan, China) at the OD value of 410 nm on an ultraviolet spectrophotometer. This was because PPO catalyzes the oxidation of phenolic compounds to quinones, which have a characteristic absorption peak at 410 nm (Steffens et al., 1994). The detection of each sample was repeated three times.

## Results

### Inheritance of Seed Coat Color

**FIGURE 1:**
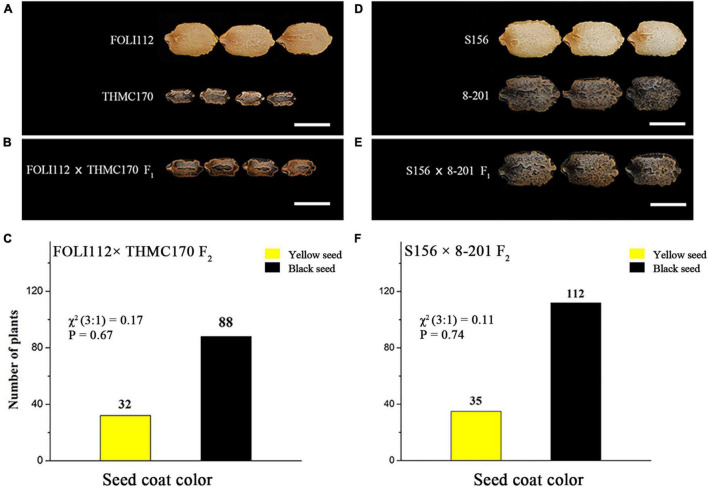
Phenotypic characterization of seed coat color in bitter gourd. **(A)** Mature seeds from intersubspecific materials FOLI112 and THMC170. **(B)** Mature seeds from FOLI112 × THMC170 F_1_ generation. **(C)** Phenotypic distribution data of seed coat colors in FOLI112 × THMC170 F_2_ population. **(D)** Mature seeds from intraspecific materials S156 and 8–201. **(E)** Mature seeds from S156 × 8–201 F_1_ generation. **(F)** Phenotypic distribution data of seed coat colors in S156 × 8–201 F_2_ population. The Chi-square (χ^2^) tests and *P*-values are displayed on **(C,F)** histograms. Bar = 1 cm.

The colors of dried seed coats from one intersubspecific hybridization, including 30 individuals each of female parent FOLI112 and male parent THMC170, 30 individuals of their F_1_ plants, and 120 individuals of their F_2_ plants, were evaluated. The results showed that seed coat colors of FOLI112 and THMC170 plants were yellow and black, respectively (Figure 1A). Moreover, all F_1_ plants exhibited a black seed coat coloration (Figure 1B). Seed coat colors of FOLI112 × THMC170 F_2_ population segregated, with 88 showing black and 32 showing yellow coloration (Figure 1C). We also evaluated the seed coat color of four progenies from an intraspecific hybridization, including 30 individuals each of female parent S156 and male parent 8–201, 30 individuals of their F_1_ plants, and 147 individuals of their F_2_ plants. Similarly, seed coat colors of S156, 8–201, and F_1_ plants showed yellow, black, and black, respectively (Figures 1D,E). The S156 × 8–201 F_2_ population was segregated, with 112 plants showing black and 35 plants showing yellow coloration (Figure 1F). The Chi-square test indicated that the black/yellow ratios of both F_2_ populations conformed to the expected ratio of 3:1 (*P* > 0.05). These results indicated that the seed coat color of bitter gourd is controlled by a single gene, named *McSC1*, making the black color dominant over the yellow color.

### Identification of the *McSC1* Locus in Intersubspecific Materials

**FIGURE 2:**
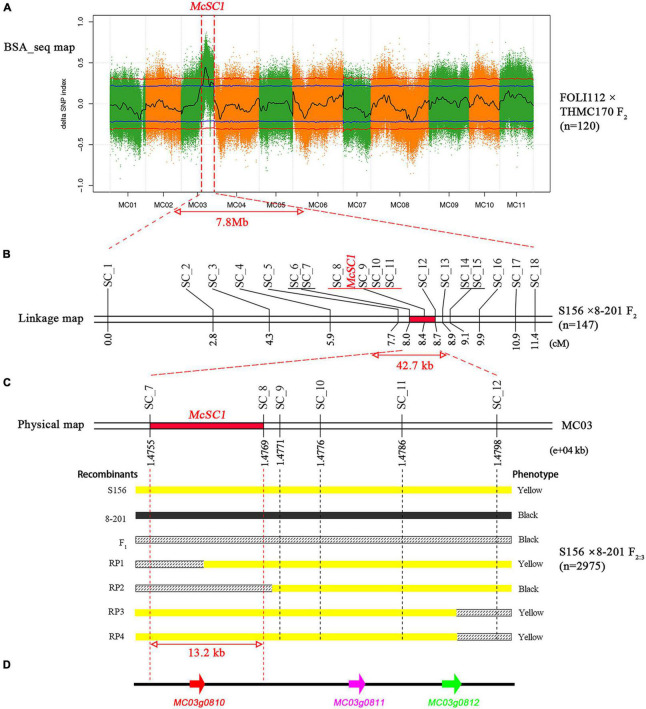
Genetic mapping of the *McSC1* locus. **(A)** BSA-seq map of the *McSC1* locus. The regions beyond the blue and red lines represent 95 and 99% confidence intervals, respectively. The region between the red dotted lines indicates the length of the *McSC1* locus. **(B)** Genetic linkage map of seed coat color. The red segment denotes the length of the *McSC1* locus. Numbers below the bar represent the genetic distance, and SC1–SC18 represent the InDel molecular markers. Genotyping data are provided in Supplementary Table 6. **(C)** Fine-mapping of the *McSC1* locus. The region between red dotted lines indicates the length of the *McSC1* locus. Numbers below the bar represent the physical position of pseudochromosome 3. RP1–RP4 represent the recombinant plants. The yellow, black, and grid bars denote the genotypes of S156, 8–201, and their F_1_, respectively. The “black” and “yellow” represent the seed coat color. **(D)** Candidate genes in the target region.

To primary map the *McSC1* locus, we conducted BSA-seq using a set of intersubspecific materials. We obtained 72.23 Gb of clean data for FOLI112, THMC170, black, and yellow DNA pools with an average of 18.06 Gb being equal to about 60 × depth per sample (Supplementary Table 3). The average Q20 value of the four samples was 95.19%, indicating a high quality of the generated resequencing data (Supplementary Table 3). Subsequently, a total of 0.02, 2.64, 2.73, and 2.74 million SNPs were identified for FOLI112, THMC170, black, and yellow DNA pools, respectively, following sequence alignment (Supplementary Table 4). After filtering, 2.43 million SNPs were concurrently obtained from the two parental lines, and the two offspring pools were selected for calculating the SNP- and ΔSNP-indexes (Supplementary Table 5). Finally, a single 95% confidence interval from 11,600,001 to 19,355,601 bp on the pseudochromosome 3, covering about 7.8 Mb in physical length, was identified based on the ΔSNP-index statistics (Figure 2A and Supplementary Table 5), and this region was inferred to be the *McSC1* locus.

### Consistent Function of *McSC1* in Intersub- and Intraspecific Materials

To confirm whether *McSC1* plays the same role in intersubspecific and intraspecific materials, we used an intraspecific S156 × 8–201 F_2_ population for linkage analysis between the seed coat color and *McSC1*. A total of 18 polymorphic InDel markers between S156 and 8–201 (Supplementary Table 2) located in a more rigorous (99%) confidence interval of the BSA-seq analysis using the intersubspecific population were developed and used to genotype the 147 individuals of the S156 × 8–201 F_2_ population. Linkage analysis indicated that the 18 markers exhibited different linkage relationships with the seed coat color (*McSC1*), ranging from 0 to 8.4 cM (Figure 2B). Among them, four markers, namely, SC_8, SC_9, SC_10, and SC_11, co-were segregated with the seed coat color in S156 × 8–201 F_2_ population (Figure 2B). Thus, based on the results of BSA-seq and linkage analysis, we inferred that the causal gene controlling the seed coat color variation in intersubspecific and intraspecific materials of the bitter gourd would be the same. Furthermore, the *McSC1* locus was delimited into a candidate interval between markers SC_7 and SC_12 with a physical length of ∼42.7 kb, ranging from 14,755,455 to 14,798,136 bp on pseudochromosome 3 (Figures 2A,B).

### Fine-Mapping of the *McSC1* Locus

A large S156 × 8–201 F_2:3_ population comprised of 2,975 seedlings was used to identify recombinant plants using two flanking markers, namely, SC_7 and SC_12. As a result, four recombinant plants (RP1–RP4) were identified. We inferred that the *McSC1* locus was delimited in a 13.2-kb region between the markers SC_7 and SC_8, following seed color observation and genotyping of the four recombinants with four co-segregating markers (Figure 2C). Based on the Dali-11 reference genome (Cui et al., 2020), we found only one protein-coding gene in this fine-mapping region, namely, *MC03g0810*, which encodes a PPO and has one intron measuring 1,570 bp and two exons measuring 482 and 862 bp in length (Figure 2D). The conserved domain analysis showed that there are three conserved domains in *MC03g0810*, including tyrosinase, PPO_DWL, and PPO_KFDV families (Supplementary Figure 1).

### Sequence Variation of *MC03g0810* in Different Bitter Gourd Germplasms

**FIGURE 3:**
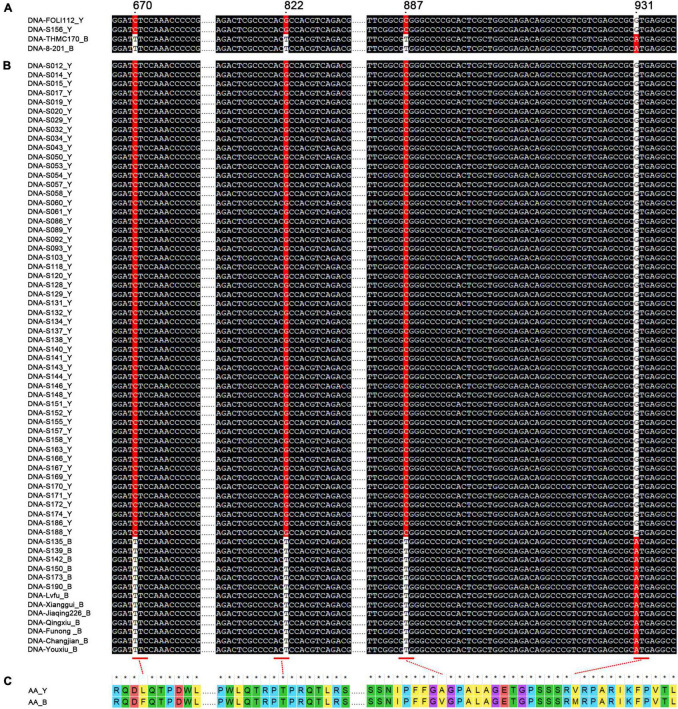
Variations of the nucleotide and amino acid sequences of *MC03g0810*. **(A)** Nucleotide mutations of *MC03g0810* in parents FOLI112, S156, THMC170, and 8–201. “Y” and “B” represent the yellow and black seed coats, respectively. **(B)** Nucleotide mutations of *MC03g0810* in 65 bitter gourd germplasms. **(C)** Conceptual amino acid mutational loci.

We obtained four SNPs, namely SNP_0810:670, SNP_0810:822, SNP_0810:887, and SNP_0810:931, within the *MC03g0810* coding region in the FOLI112, S156, THMC170, and 8–201 parental lines (Figure 3A). These four SNPs formed two haplotypes embodying C–G–C–G and T–T–T–A (in the order: SNP_0810:670, SNP_0810:822, SNP_0810:887, and SNP_0810:931). The C–G–C–G haplotype (FOLI112 and S156) had yellow seed coats, while the T–T–T–A haplotype (THMC170 and 8–201) displayed black seed coats. Additionally, we sequenced and compared *MC03g0810* among 58 + 7 inbred lines (seven F_1_ hybrids with homozygous *MC03g0810*) exhibiting distinct genetic backgrounds and 24 F_1_ hybrids containing heterozygous *MC03g0810* to evaluate the relationship between the haplotype and the seed coat color. The results showed that all the 52 inbred lines with yellow seed coats were C–G–C–G haplotypes, and 13 inbred lines with black seed coats were T–T–T–A haplotypes, which was consistent with previous results (Figure 3B and Supplementary Table 1). Meanwhile, the 24 F_1_ hybrids were heterozygous for the C–G–C–G and T–T–T–A haplotypes (Supplementary Table 1). Generally, the variation of the seed coat color in different bitter gourd germplasms correlates with the *MC03g0810* haplotype. Moreover, the four SNPs of *MC03g0810* generated two types of protein sequences corresponding to the two haplotypes (Figure 3C).

### Characterization of *MC03g0810* Expression and Polyphenol Oxidase Activity

**FIGURE 4:**
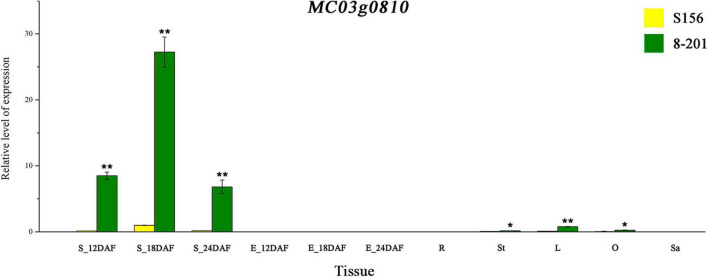
Expression pattern of *MC03g0810*. Yellow and green bars indicate the gene expression levels in S156 and 8–201, respectively. DAF denotes the day after flowering, and S_12DAF, S_18DAF, and S_24DAF represent the seed coats at 12DAF, 18DAF, and 24DAF, respectively. The letters E, R, St, L, O, and Sa represent the embryo, root, stem, leaf, ovary, and sarcocarp, respectively. Error bars indicate the standard errors (SE). *P < 0.05 and **P < 0.01 using Student’s *t*-test.

The results of tissue-specific expression analysis showed that *MC03g0810* had different expression levels in the seed coat, leaves, stems, and ovaries; however, it was barely expressed in embryos, roots, and sarcocarps (Figure 4). Furthermore, the *MC03g0810* expression levels in the seed coat were significantly higher in three developmental stages (12 DAF, 18 DAF, and 24 DAF) of 8–201 than in S156 (Figure 4). These results suggest that *MC03g0810* has a relatively higher tissue specificity and shows the highest expression level at the 18 DAF stage of black seed coats, indicating that *MC03g0810* is associated with the black seed coat.

**FIGURE 5:**
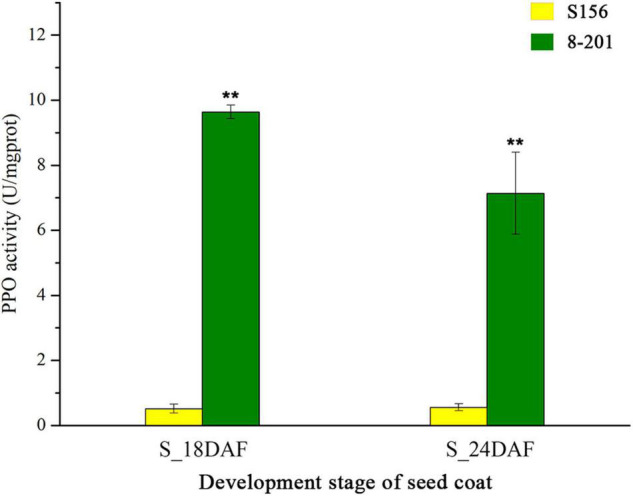
Polyphenol oxidase activities of seed coats of bitter gourd. Yellow and green bars indicate the PPO activities in S156 and 8–201, respectively. The S_12DAF and S_18DAF represent the seed coats at 18 and 24 days after flowering (DAF), respectively. The error bars indicate standard errors (SE). **P < 0.01 using Student’s *t*-test.

The PPO-induced browning is a major path darkening color in various plant tissues (Yoruk and Marshall, 2003). Therefore, considering that *MC03g0810* is a member of the PPO gene family, we compared the PPO activity of seed coats at 18 and 24 DAF between the S156 and 8–201 lines. Results showed that the PPO activity of the 8–201 seed coat was approximately 19.2 and 12.8 times higher than that of the S156 seed coat at 18 and 24 DAF, respectively (Figure 5). This was consistent with the differential expression of *MC03g0810* in the seed coats of S156 and 8–201. We speculate that the formation of the black seed coat in 8–201 may be correlated with higher PPO activity, resulting from the higher expression of *MC03g0810*.

## Discussion

The color variation of the yellow and black seed coats was observed in both cultivated and wild bitter gourd germplasms (Kole et al., 2020). Previous studies have established that the seed coat color is controlled by a single gene in the bitter gourd and that the black color is dominant over the yellow color in intersubspecific and intraspecific materials (Liu, 2011; Tan et al., 2013). Given this fact, we simultaneously used two F_2_ segregating populations from two respective intersubspecific and intraspecific hybridizations for the genetic analysis of the seed coat color. The results were consistent with those of previous studies (Figure 1). However, it is not clear whether these two genes from different genetic backgrounds are the same.

With the rapid development and remarkably decreasing cost of sequencing technologies, whole genome sequencing and resequencing of bitter gourd had been completed (Urasaki et al., 2017; Cui et al., 2020; Matsumura et al., 2020), which laid a solid foundation for gene mapping. Accordingly, we identified the *McSC1* locus related to the seed coat color in an intersubspecific F_2_ population using BSA-seq (Figure 2A). Moreover, linkage analysis verified that this *McSC1* locus is also related to the seed coat color in an intraspecific F_2_ population (Figure 2B). These studies provide evidence that the causal gene responsible for the seed coat color is the same in intersubspecific and intraspecific bitter gourd germplasms.

In addition, studies have suggested that dominance of the black seed coat coloration of bitter gourd over the creamy color conforms to the digenic inheritance mode, of which only one of two genes was mapped on LG3 between the AFLP markers, namely, E12M47a and E11M48a (Kole et al., 2012). Due to the dearth of information on E12M47a and E11M48a sequences, we failed to compare the locus of these sequences with the *McSC1* locus. However, we detected the same digenic inheritance pattern in some other bitter gourd materials, indicating that there may be other genes regulating the formation of black seed coats in the bitter gourd (not published).

To scale down the probable size of the *McSC1* locus, we conducted fine-mapping in a large intraspecific F_2:3_ population. As a result, the *McSC1* locus was further fine-mapped to a 13.2-kb region (Figure 2C), much narrower than the ∼300 kb determined by Cui et al. (2022) *via* a genome-wide association study. By comparing it to the Dali-11 reference genome (Cui et al., 2020), we found that the 13.2-kb region contained only one candidate gene, *MC03g0810*, encoding a PPO protein. Therefore, it is evident that the forward genetics strategy provides the most direct evidence, proving that *MC03g0810* is the causal gene of *McSC1* in bitter gourd.

We cloned the *MC03g0810* genes of 89 germplasms from different genetic backgrounds, including inbred lines and commercial F_1_ hybrids, to further understand the relationship between the variation of *MC03g0810* and the seed coat color in different bitter gourd materials. As a result, we detected two haplotypes, namely, C–G–C–G and T–T–T–A, whereby C–G–C–G was contained in germplasms with yellow seed coats, while T–T–T-A was only found in the germplasms with black seed coats (Figure 3B and Supplementary Table 1). A haplotype is a combination of a series of genetic variation loci coexisting on the same chromosome and is an essential aspect of genetic research (Fan et al., 2011; Snyder et al., 2015). Thus, identifying *MC03g0810* haplotypes will help understand the relationship between different SNP combinations and the seed coat color. It is also crucial in determining a suitable marker-assisted selection for the *MC03g0810*-mediated seed coat color in bitter gourd breeding programs.

Among the four *MC03g0810* SNPs, three (SNP_0810:670, SNP_0810:887, and SNP_0810:931) are non-synonymous, while the remaining one (SNP_0810:822) exhibits synonymous mutations (Figure 3C). Furthermore, the conserved domain analysis suggested that SNP_0810:822, SNP_0810:887, and SNP_0810:931 are located in the non-conservative domain region, and only SNP_0810:670 belonged to the conserved DWL domain of the PPO protein (Supplementary Figure 1). Therefore, the SNP_0810:670 mutation, involving the substitution of leucine (L) with phenylalanine (F), should be focused on, since it may be the causal mutation that downregulates the expression of *MC03g0810* and thus reduces the PPO activity, resulting in the seed coat color changes (Figure 3).

Polyphenol oxidases are copper metalloenzymes widely existing in plants and are involved in the browning of plant tissues (Yoruk and Marshall, 2003; Zhang and Sun, 2021). Previous studies have shown that mutations of the *PPO* gene, which darken the seed coat color, exist in rice (Yu et al., 2008), wheat (Beecher and Skinner, 2011; Beecher et al., 2012), and barley (Taketa et al., 2010), among other crops. Recently, a similar PPO-mediated mechanism has been reported in Cucurbitaceae; for example, *Cla019481* encoding a PPO protein was considered a candidate gene of *ClCS1* for the black seed coat color in watermelon (Li et al., 2020). Spatial–temporal expression patterns of *Cla019481* and *MC03g0810* (Figure 4) and variations in the PPO activities of seed coats (Figure 5) exhibited high similarities between watermelon and bitter gourd. These results suggest that the regulatory mechanisms of seed coat color blackening may be consistent in watermelon and bitter gourd, thus providing a reference for studies on the seed coat color in other cucurbits. Notably, according to the Dali-11 reference genome of bitter gourd (Cui et al., 2020), *MC03g0810* and the other three paralogs of *PPO* genes, including *MC03g0811*, *MC03g0812*, and *MC03g0813*, are adjacent in their physical positions, forming a *PPO* cluster. The *PPO* clusters have been reported in rice, tomato, and red clover (Newman et al., 1993; Winters et al., 2009); however, there is no report on *PPO* clusters in Cucurbitaceae so far. Our expression analyses suggested that the *PPO* genes have similar expression patterns with *MC03g0810* in seed coats (not published). Therefore, more efforts are needed to determine whether there exists a relationship between the other three *PPO* paralogs and the seed coat color in bitter gourd.

## Conclusion

The seed coat color of bitter gourd is controlled by a single gene named *McSC1*. The *McSC1* locus was fine-mapped to a 13.2-kb region on pseudochromosome 3, which contains only one candidate gene, *MC03g0810*, encoding a polyphenol oxidase protein. Based on the sequence, expression, and PPO activity analyses, *MC03g0810* was considered the causal gene of *McSC1*.

## Data Availability Statement

The datasets presented in this study can be found in online repositories. The name of the repository and accession number can be found below: National Center for Biotechnology Information (NCBI) BioProject, https://www.ncbi.nlm.nih.gov/bioproject/PRJNA808187.

## Author Contributions

JZ, JWC, and KH conceived, designed all experiments, and wrote the manuscript. JZ, JD, YZ, and JL performed the experiments. JZ, JJC, and FH analyzed the data. All authors read and approved the final manuscript.

## Conflict of Interest

The authors declare that the research was conducted in the absence of any commercial or financial relationships that could be construed as a potential conflict of interest.

## Publisher’s Note

All claims expressed in this article are solely those of the authors and do not necessarily represent those of their affiliated organizations, or those of the publisher, the editors and the reviewers. Any product that may be evaluated in this article, or claim that may be made by its manufacturer, is not guaranteed or endorsed by the publisher.

## Footnotes

1
https://www.ebi.ac.uk/Tools/msa/clust alo/

2
https://www.ncbi.nlm.nih.gov/Structure/cdd/wrpsb.cgi
