# Supplementary material for: MC03g0810, an Important Candidate Gene Controlling Black Seed Coat Color in Bitter Gourd (Momordica spp.)
Source: Front Plant Sci. 2022 Apr 27;13:875631. doi: 10.3389/fpls.2022.875631 (PMC9094142; doi:10.3389/fpls.2022.875631)
Supplement: Supplementary file 1 [file Table_1.DOCX]

Supplementary Material

## Supplementary Figures
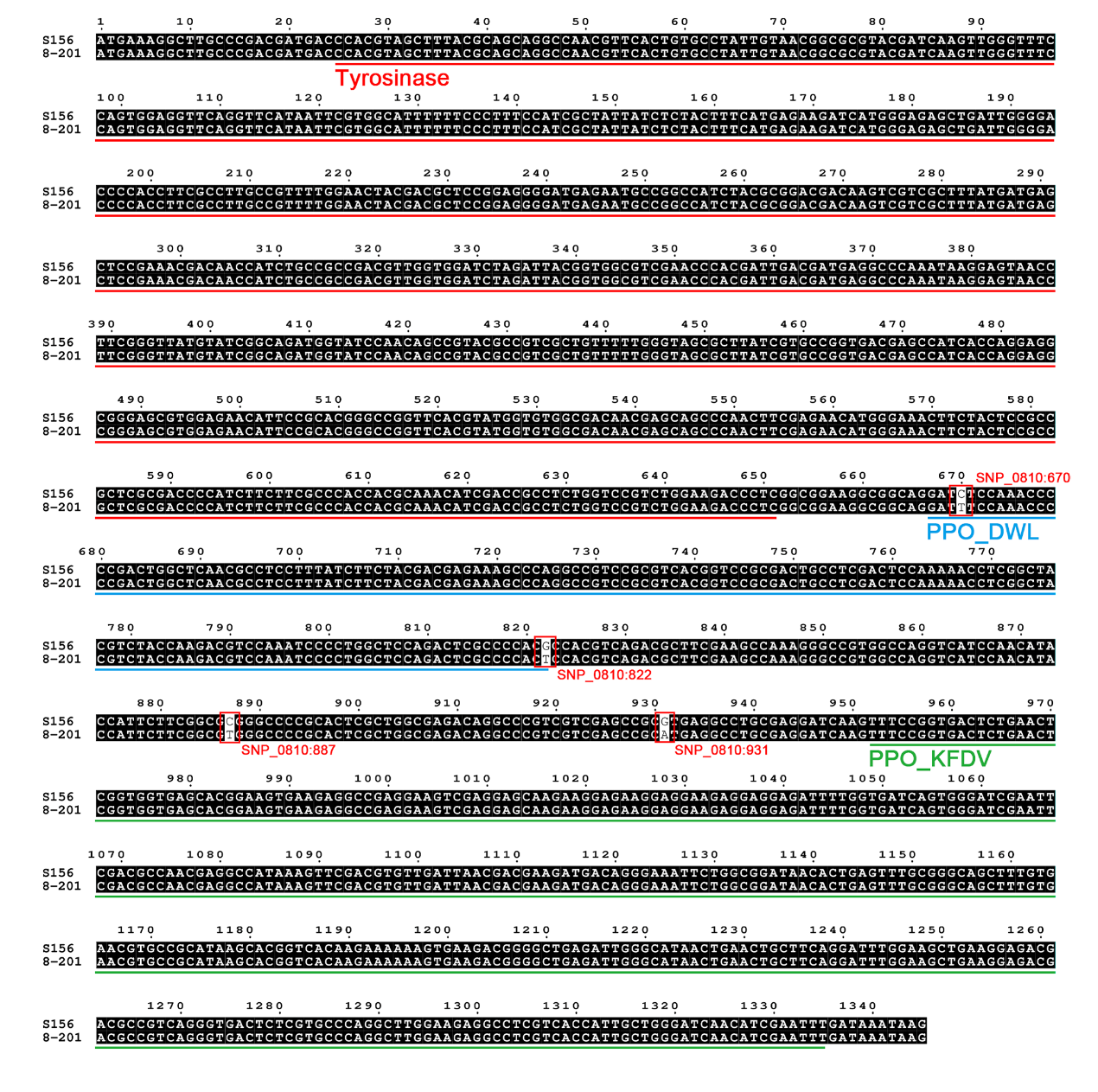


**Supplementary Figure 1.** Conserved domain layout of *MC03g0810*. Red, blue, and blue straight lines represent Tyrosinase, PPO_DWL, and PPO_KFDV conserved domains, respectively. Red boxes denote Nucleotide mutations of MC03g0810 between S156 and 8-201.
