# Supplementary figures and images for: MC03g0810, an Important Candidate Gene Controlling Black Seed Coat Color in Bitter Gourd (Momordica spp.)
Source: Front Plant Sci. 2022 Apr 27;13:875631. doi: 10.3389/fpls.2022.875631 (PMC9094142; doi:10.3389/fpls.2022.875631)

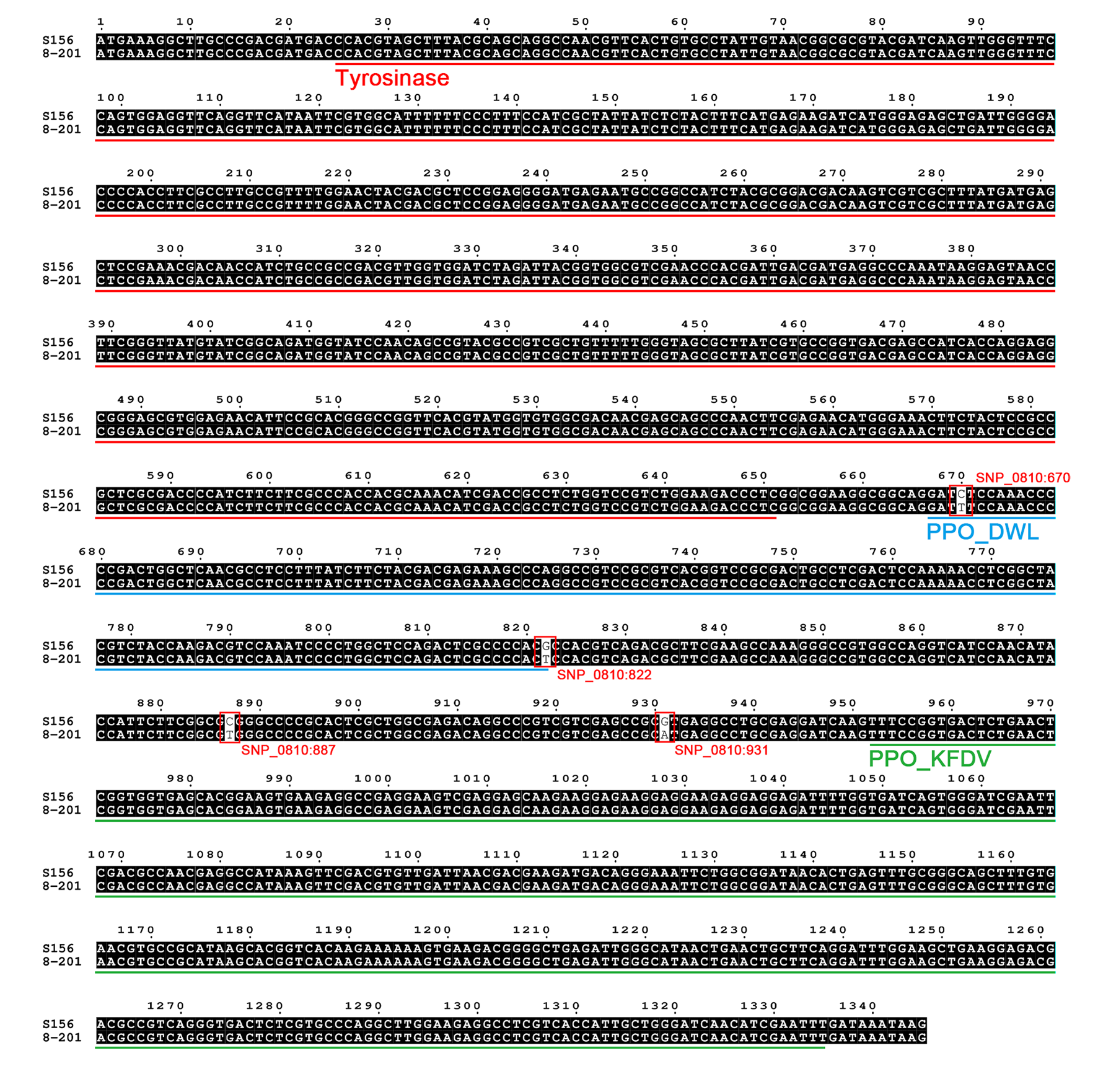

Supplement: Supplementary file 3 [file Image_1.TIF]
